# Latent Classes of Adverse Childhood Experiences (ACEs) and Adult Substance-use Problems and Psychosocial Outcomes: Complex and Heterogeneous Associations

**DOI:** 10.1007/s11469-025-01527-w

**Published:** 2025-08-06

**Authors:** Emily S. Herberholz, Rachel E. Wolchok, Jeffrey M. Rogers, Sophia Selenou-Yemgang, Bianca D. Beckwith, Kirsten E. Smith, David H. Epstein

**Affiliations:** 1 Real-world Assessment Prediction & Treatment Unit, National Institute on Drug Abuse Intramural Research Program, 251 Bayview Blvd, Baltimore, MD 21224 USA; 2 Department of Pharmacology and Behavioral Sciences, Johns Hopkins University School of Medicine, Baltimore, MD USA; 3 Department of Biology, Morgan State University, Baltimore, MD USA

**Keywords:** Adverse childhood events, Latent class analysis, Adult psychological symptoms, Substance use disorder, Substance use patterns, Adult outcomes

## Abstract

Adverse childhood experiences (ACEs), expressed as summary scores, are associated with adverse adult outcomes. We aimed to detect more nuanced associations via latent class analysis (LCA) of ACEs. We tested associations between class membership and adult anxiety, depression, loneliness, stress, and substance-use patterns/disorders. Our nationwide data set, part of our larger study of substance use with or without addiction, included 2615 mTurk workers with any past-6-month alcohol use (71.6% *n* = 986) or any past-6-month nonmedical use of opioids or stimulants (28.4%, *n* = 391). Using a 10-item ACE questionnaire, we identified high, moderate, and two differentiable low-ACE classes (exposed to either parental substance use or divorce). In regression models, the high-ACE class had more past-year substance use (ORs 2.01–3.38, *R*^2^ = 0.12), problematic use (ORs 1.89–2.66, *R*^2^ = 0.10), SUD treatment (ORs 2.10–5.45, *R*^2^ = 0.15), and psychological symptoms (betas 1.32–8.74, *R*^2^ = 0.11–0.19). Despite aggregate associations, the low *R*^2^ values underscore within-class heterogeneity in outcomes. ACE patterns are informative about adult outcomes in the aggregate, but do not support case-by-case inferences.

**Supplementary Information:**

The online version contains supplementary material available at 10.1007/s11469-025-01527-w.

## Background on Adverse Childhood Experiences

Adverse childhood experiences (ACEs)—a constellation of stressors occurring in children ages 0–17 including abuse, neglect, and household violence—are prevalent in the USA; in one recent survey, 64% of adults reported some experience of ACEs (CDC, 2024). ACEs were first operationalized by Felitti et al. (1998) who proposed seven initial categories: psychological, physical, or sexual abuse; violence against mother; or living with household members who were substance misusers, mentally ill or suicidal, or ever incarcerated. The conceptualization of ACEs resulted in decades of exploration into the longitudinal relationship between early life stressors and mental and physical health outcomes. These exposure/outcome associations have been variously attributed to disruptions in cognitive styles or emotion regulation, with accompanying maladaptive behaviors (Helitzer et al., 2015; Liu et al., 2013; Sachs-Ericsson et al., 2006) or to biological mediators such as disrupted white-matter integrity (Benedetti et al., 2014) or allostatic wear on multiple bodily system (Danese & McEwen, 2012). In the present study, we focus on the exposures and associations themselves, not on putative mechanisms.

Below, we note two areas of contention in ACE literature—one concerning scoring methods and the other concerning the strength of associations between ACEs and life outcomes.

### Methods of Scoring ACEs

Traditionally, ACE exposure has been quantified in one of two ways: by individual categories of events or by cumulative risk scores summing all categories. Using the former approach, single adversities such as sexual abuse (Fergusson et al., 2013) and physical abuse (Lansford et al., 2021) have been postulated as potential precursors for mental and physical health problems later in life. One drawback of focusing on individual ACE categories is that it does not acknowledge ACEs as multiplexes; one in six adults report experiencing four or more types of ACEs (CDC, 2021), and experiencing one ACE category increases the likelihood of experiencing at least one more (Dong et al., 2004).

In their seminal work, Felitti et al. (1998) recognized the co-occurrence of ACEs through a cumulative risk (CR) approach. The CR approach involves the summation across categories to create a total score of ACEs from 0 to 7. The authors observed a graded dose–response relationship between breadth of ACE exposure and later experiences of substance-use disorders, depression, and other negative outcomes. These findings have been replicated and extended to such outcomes as anxiety (Almuneef et al., 2016), depression (Almuneef et al., 2016; Chapman et al., 2004), and suicidality (Y.-R. Wang et al., 2019).

The CR approach has limitations of its own: by simply summing ACE categories, it fails to consider that ACEs should not all be weighted equally. It also implicitly assumes that all ACEs influence later life outcomes through the same underlying mechanisms (McLaughlin & Sheridan, 2016).

These limitations can be addressed by statistical approaches such as latent-class analysis (LCA), a person-centered approach in which survey respondents or study participants are grouped into “classes” reflecting their patterns of ACE exposure. (When exposures are expressed as numerical scores rather than presence versus absence, they can be classified with a similar approach called latent-profile analysis, LPA.) Class assignment in LCAs can reflect both overall frequencies of exposures and specific co-occurrences of exposures. LCA can provide insight into associations between such co-occurrences and different life outcomes (Lanier et al., 2018).

In a retroactively assessed LPA of a community sample of adult responses to an ACE questionnaire (Dong et al., 2004; Felitti et al., 1998), four profiles were identified based on 10 categories of ACEs (the seven categories listed above, plus emotional neglect, physical neglect, and parental divorce/separation) (Dobson et al., 2021). The four profiles were labeled dysfunctional family environment, sexually abused, emotionally abused, and no mistreatment. Profile membership was differentially related to patterns of mental-health outcomes in adulthood. For example, people in the emotionally abused profile were higher in anxiety than people in the dysfunctional family environment and sexually abused profiles, while people in the no mistreatment profile were lower in anxiety than people in the three profiles. These findings suggest that a profile-based or class-based approach to quantifying ACE exposure is useful for investigating how ACEs predict later-life outcomes.

### ACE Influence on Later-Life Outcomes

Another realm of uncertainty, however, is the extent to which ACE/outcome associations can be stated as more than group-level associations. For example, Cavanaugh et al. (2015), using LCA with ACEs data from the National Epidemiologic Survey on Alcohol and Related Conditions (NESARC), found associations between membership in four ACE classes (high/multiple ACEs, high parental substance abuse, high childhood physical abuse, and low ACEs) and classes of later psychiatric diagnoses, including diagnoses of substance-use disorders. But more than a third of people in the high/multiple ACEs profile were in the outcome class reflecting few or no diagnoses. The authors interpreted this finding to suggest that many people are resilient to ACEs (we will return to the concept of resilience in the “Discussion” section). Baldwin et al. (2021) used data from two population-based birth cohorts (*N* = 2927) to examine how well ACEs can predict individual risk for later health problems. Prospectively, across 20 years, in both cohorts, ACE scores were associated with increased likelihoods of adult physical and mental health problems at a group level—but could not provide accurate person-level predictions.

### Present Study

As part of a broader study of substance use and psychological and social functioning, we collected ACEs data from US adults with any past-6-month alcohol use (whether problematic or not) or any past-6-month nonmedical use of opioids or psychostimulants (whether problematic or not). Prior research has shown strong group-level associations between summed ACE-exposure scores and the likelihood of cigarette use (Anda et al., 1999), alcohol use (Dube et al., 2006), and other drug use (Dube et al., 2003), but has not used LCA to clarify the contribution of specific ACE *combination*s, especially with regard to substance-use disorders (SUDS) (Leza et al., 2021). We used LCA and multiple regression to address the following research questions in our sample: (1) What are the identifiable latent classes of ACE exposure? (2) Is ACE class membership associated at group level with adult substance-use patterns, SUDs, and psychological symptoms (i.e., anxiety, depression, loneliness, and stress)? (3) If so, then how well do those group-level associations support person-level predictions about ACE exposure and later outcomes? Although some of our questions center on methodology, the answers should help determine conclusions and clinical recommendations.

## Method

### Screening and Recruitment

These data were drawn from a larger survey study examining adverse social conditions and substance use in US adults (Rogers et al., 2022; Smith et al., 2022). The original study was conducted on the online crowdsourcing platform Amazon Mechanical Turk (MTurk), which is a widely used tool in behavioral science research. Recent investigations have shown large numbers of mTurk “workers” are bots and/or provide inconsistent data. We utilized MTurk filters and IDs, multi-stage quantitative and qualitative screening, and survey software attention/validity checks (described in the “Data Collection” section) to increase confidence in data quality.

Respondents were eligible if they met the following criteria: a) 18 years of age, b) US resident (verified by IP address and VPN detection), c) English language proficiency, and who had completed > 100 MTurk human intelligence tasks (HITs). Eligible respondents were those who reported substance use within 6 months prior to screening that fell under one of the following categorizations: a) alcohol but no other substances, except for nicotine and caffeine, or b) at least one psychostimulant or opioid substances in the past 6 months, allowing all other substances. These groups were specified in recruitment to examine differences in experiences, attitudes, mental health, and SUDs among people with more conventional substance use only (alcohol, caffeine, etc.) and those who use more stigmatized substances (opioids and psychostimulants; Buchman et al., 2017; Flores et al., 2023; Wakeman & Rich, 2018).

### Data Collection

Detailed study recruitment information is reported in prior manuscripts examining these data (Rogers et al., 2022; Smith et al., 2022). Of the 13,608 screening surveys, 3417 (25.1%) met inclusion for past 6-month alcohol use group and 1695 (12.5%) for past 6-month opioid-psychostimulant use group (inclusive of kratom usage). Those who completed the full Qualtrics survey received $7.25 in compensation. Programmed data quality checks were a) consistency of responding across demographic and substance use questions between screening and survey data, b) detection of unitary or atypical responding across measures, and c) endorsement of nonsense items (e.g., “Are you allergic to bananafish?”). Respondents were unenrolled if they failed three quality checks or exceeded the 4-h completion window. Completed responses were identified by MTurk worker ID and IP address to ensure that only one survey was completed by each person. IP addresses were scanned by proxycheck (proxycheck.io, 2018/2024) for proxy or VPN addresses. No personally identifiable information was collected beyond IP addresses, later deleted following VPN checks, and the study was exempt from the National Institutes of Health Intramural Research Program IRB.

From the 5112 MTurk workers eligible for the study, 3261 (63.8%) began the survey with 2864 (56.0%) completing it. A total of 249 responses were removed as a result of an IP address-related exclusion (i.e., duplicated, indeterminate, or sourced outside of the USA), discrepancies between screener and survey responses regarding inclusion criteria, or improbably brief survey completion times. The final sample population comprised 2615 complete responses, with 1630 (62.3%) in the past 6-month alcohol/tobacco group and 985 (37.7%) in the past 6-month use of at least one opioid or stimulant group.

### Measures

#### Demographic Characteristics

Demographics were assessed through 14 questions related to age, body height/weight, education, employment, sexual orientation, geographic location, income, legal involvement, and parental status.

#### Adverse Childhood Experiences

Adverse childhood experiences (ACEs) were measured using the Adverse Childhood Experiences questionnaire, which comprised 10 items relating to experiences of social dysfunction, abuse, neglect, and other stressors during the first 18 years of life (Dong et al., 2004). Item responses were entered individually as response variables in LCA models.

#### Psychosocial Assessments

The survey included measures of depressive symptoms, anxiety symptoms, general stress, and loneliness. The 7-item Generalized Anxiety Disorder Scale (GAD-7; Delgadillo et al., 2012; Löwe et al., 2008) assessed past-month anxiety symptom experiences. The 10-item Center for Epidemiologic Studies Short Depression Scale (CES-D-R-10) (Björgvinsson et al., 2013; Miller et al., 2008) assessed past-30-day depressive symptoms. The 20-item UCLA loneliness scale (Cacioppo et al., 2006; Russell, 1996) assessed current feelings of loneliness. The 14-item Perceived Stress Scale (Cohen et al., 1983) assessed past-30-day stress/distress and coping. These measures displayed good to excellent internal consistency in this sample, as indicated by Omega total coefficients (McNeish, 2018): Perceived Stress Scale *ω*_Total_ = 0.85, CES-D-R-10 *ω*_Total_ = 0.91, GAD-7 *ω*_Total_ = 0.93, UCLA loneliness scale *ω*_Total_ = 0.96. Total unadjusted measure scores were used in data analyses.

#### Substance-Use Assessment

Participants were asked whether they had experienced problems related to substance use and to indicate which, if any, substance had caused them the most problems in the past year. Participants were then administered a checklist of DSM-5 criteria for SUD for that substance, or, if no substance was named as problematic, the same checklist was used for the substance they reported having using most frequently over the past year. Participants were also asked to indicate whether they had ever received treatment for substance-use problems.

### Statistical Analyses

#### Latent-Class Analysis (LCA)

To identify latent types or “classes” of ACEs, we modeled participants’ ACE questionnaire response using latent class analysis (LCA) models, which are fit on binary or categorical variables using maximum likelihood estimation. LCA models randomly split observations into a specified number of groups and reassign observations based on increasing model maximum likelihood. This process continuous until the best-fitting set of classifications is found. Because ACE questionnaire responses are dichotomous (yes/no), no variable transformations were required for modeling. All statistical analyses were conducted using R (4.2.2; R Core Team, 2024) and followed best-practice recommendations from field experts and LCA-software authors (Larose et al., 2016; Nylund-Gibson & Choi, 2018; Vermunt, 2010; Weller et al., 2020), save for software-necessitated deviations in analysis of latent class covariates. R package *poLCA* (Linzer & Lewis, 2011) was used to fit LCA models. Quantitative indices for identifying the best-fitting class solution were Bayesian information criterion (BIC), model-parameter-adjusted BIC (aBIC), and sample size-corrected Akaike information criterion (cAIC), for which lower values indicate a better-fitting model. After identifying the ideal number of classes, we examined model suitability in terms of model entropy (relative entropy criterion, a measure of how well the model assigns observations to classes) and average class posterior probabilities, which provide distinct sources of evidence for model assignment accuracy. Current heuristic recommendations state that model entropy and average class posterior probabilities should exceed at least 0.80 to be considered minimally appropriate for further examination, with > 0.90 being ideal (Weller et al., 2020). Because software authors suggest running multiple model iterations with variable repetitions to ensure that a global maximum likelihood has been identified, LCA models were fit using 20, 30, 40, and 50 algorithmic repetitions at each class specification level (e.g., 1-class, 2-class, etc.) to ensure that the indicated number of classes did not vary by selected number of algorithmic repetitions. To generate qualitative descriptions of the four identified latent classes, we examined the latent classes’ conditional item-response probabilities (referred to as “item probabilities” [Pc =]), which are the estimated probabilities of ACE-item endorsements made by a given member of each latent class. Because LCA models are probabilistic, not every observation within a class will display all characteristics of that class.

Following latent-class-modeling procedures, probabilistic class assignments were extracted, and we proceeded with analysis of between-class differences in demography, past-year substance use, and SUD. Best practices for this procedure (see LCA “3-step procedures” from Vermunt, 2010) are not currently feasible in R. To accommodate this, we fit generalized linear models that controlled for LCA model classification uncertainty and which used simple between-class contrasts. Between-class differences in demographic factors were examined first, and all subsequent models covaried for demographic factors that significantly differed between classes. Final models covaried for sex, educational attainment, annual income level, employment status, and perceived childhood socioeconomic status. Binomial models were used for dichotomized response variables (e.g., past-year use) and for expressing the likelihood of belonging to one demographic group over another; Gaussian models were used for the CES-D-R-10, GAD-7, Perceived Stress Scale, and UCLA Loneliness Scale scores; zero-inflated negative-binomial models were used for counts of DSM-5 SUD symptoms. We provide estimates of effect size as odds ratios (OR) in binomial and zero-inflation models, partial *r*^2^ in Gaussian models, and incidence rate ratios (IRR) in count models.

## Results

### LCA Model Results

**Fig. 1:**
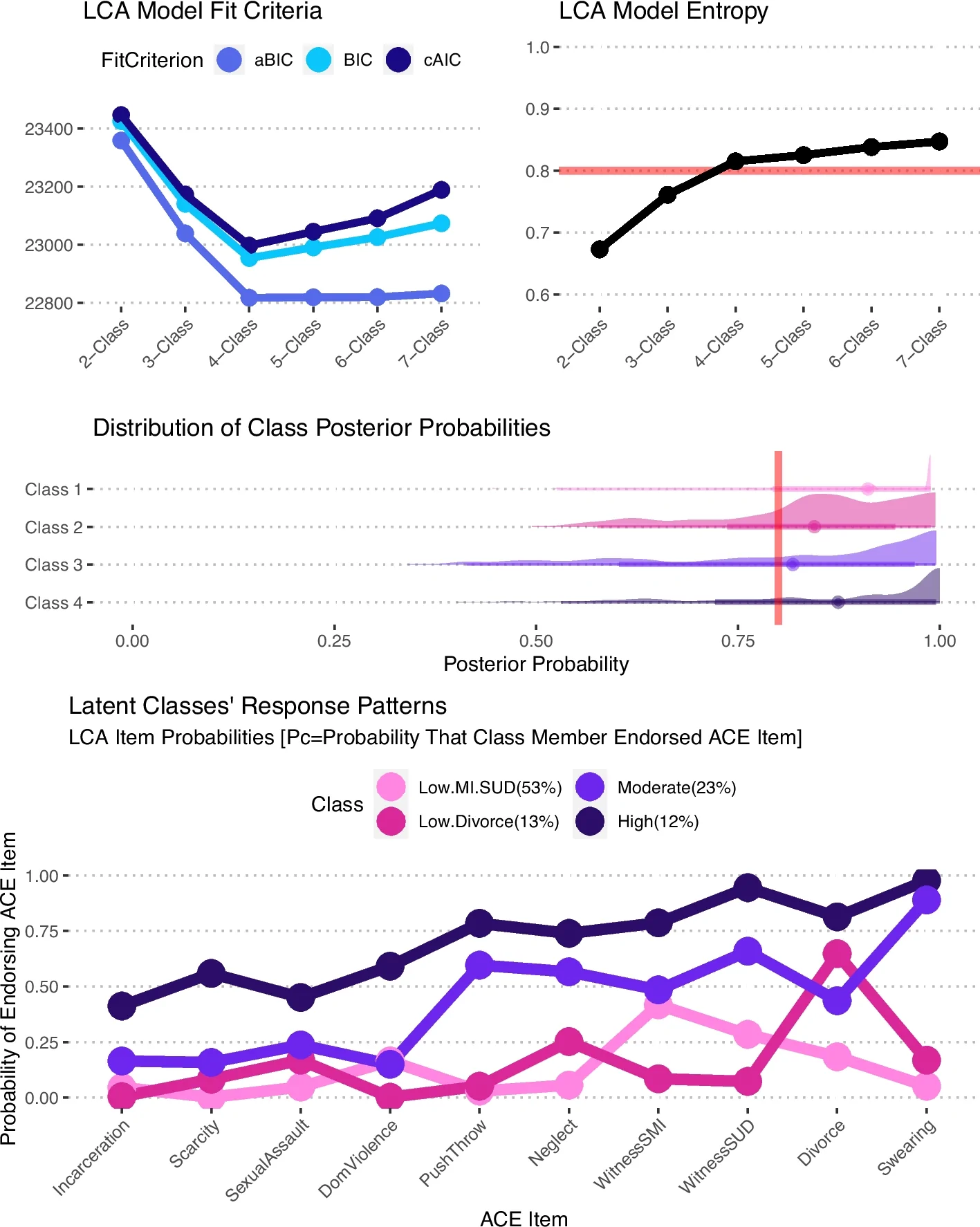
Latent-class analysis: model-fit indices (top left panel), entropy (top right panel), and posterior probability (middle panel) of class assignment. LCA model-fit indices are Bayesian information criterion (BIC), parameter-adjusted BIC (aBIC), and sample-size-corrected Akaike information criterion (cAIC). Bottom panel: Latent-class-analysis response pattern shown in terms of item probabilities, which may be interpreted as the probability that a member of a given latent class endorsed a given ACE item

A 4-class model specification was found to provide the best fit for the data as evidenced by lowest values for BIC, aBIC, and cAIC. The relative entropy criterion was 0.81, exceeding the 0.80 heuristic recommendation. Average class posterior probabilities were all greater than the recommended minimum threshold (= 0.80): class 1 = 0.91, class 2 = 0.84, class 3 = 0.82, class 4 = 0.87. Figure 1 shows an elbow plot of model-fit criteria from latent-class models specifying one through seven latent classes (top left panel), an elbow plot of LCA models’ entropy criteria (top right panel), distributions of latent-class posterior probability of assignment for the 4-class model (middle panel), and conditional item probabilities for each class (bottom panel). Item probability (Pc =) may be interpreted as the conditional probability that a given member of a given latent class endorsed an ACE item.

### Qualitative Description of Latent Classes

*Class 1—Low ACES except parental mental illness or SUD (low-MI-SUD).* This class represents 53% of the overall sample and is best characterized by low probability of most ACEs (Pc = 0.001 to 0.18), except for their being more likely than class 2 to report having caretakers with mental illness (Pc = 0.41 vs. Pc = 0.08) or caretakers with an SUD (Pc = 0.28 vs. Pc = 0.07).

*Class 2—Low ACES except parental divorce (low-divorce).* This class represents 13% of the overall sample and is best characterized by low probability of most ACEs (Pc = 0.001 to 0.17), except for their being more likely than class 1 to report experiencing divorce (Pc = 0.65 vs. Pc = 0.18) and neglect (Pc = 0.25 vs. Pc = 0.06).

*Class 3—Moderate ACES.* This class represents 23% of the overall sample and is best characterized by a mixture of high and low probabilities of ACEs. This class was more likely than the two low-ACE classes to report experiences of physical aggression (Pc = 0.60), neglect (Pc = 0.57), caretakers with mental illness (Pc = 0.49), caretakers with an SUD (Pc = 0.66), divorce (Pc = 0.44), and verbal aggression (Pc = 0.89). This class’s item response probability falls in between the low classes and the high class on most items; they were less likely than low-divorce to report divorce (Pc = 0.44 vs. Pc = 0.65) and were similar to low-MI-SUD in likelihood of having caretakers with mental illness (Pc = 0.49 vs. Pc = 0.42).

*Class 4—High ACES.* This class represents 12% of the overall sample and is best characterized by its high probability of endorsing every ACE item, relative to all other classes. These differences were especially pronounced in endorsement of witnessing domestic violence (Pc = 0.59), caretaker incarceration (Pc = 0.41), resource scarcity (Pc = 0.56), and sexual assault (Pc = 0.45)—items for which all other classes had probabilities of 0.25 or lower.

### Demographics

Descriptive statistics by ACE class are in Table 1. The sample was, on average, 36.7 +/− 11.3 years old, 59.8% female identified, and 78.7% White; 54.2% had at least 4 years of college, and 63.7% were employed full time or were students. We examined demographic differences across ACE classes using binomial-regression models. There were no significant age differences, with the largest difference being 1.19 years. Members of the low-divorce (OR = 1.49), moderate (OR = 1.56), and high (OR = 1.70) classes were more likely to identify as female than the low-MI-SUD class. There was no significant association between class membership and race/ethnicity. Compared to the low-MI-SUD class, the low-divorce (OR = 0.39), moderate (OR = 0.54), and high (OR = 0.26) classes were less likely to have obtained a 4-year college degree. Annual income was defined as either above or below poverty line, which was calculated using US state of residence, marital status, and number of children in home to contextualize income. There was no significant difference in annual income between low-ACE classes, but the moderate (OR = 1.38) and high (OR = 1.51) classes were more likely to be earning below poverty line annual incomes than the low-MI-SUD class. Similarly, compared to the low-MI-SUD class, the moderate (OR = 1.84) and high (OR = 2.12) were more likely to be unemployed and looking for work or disabled.

**Table 1:** Sample demographic and descriptive statistics, split by LCA class and for the overall sample

	Low-MI-SUD (*n* = 1377, 53%)	Low-divorce (*n* = 331, 13%)	Moderate (*n* = 589, 23%)	High (*n* = 318, 12%)	Total (*N* = 2615)
Study recruitment group					
Past-6-month alcohol/tobacco	986 (71.6%)	179 (54.1%)	334 (56.7%)	131 (41.2%)	1630 (62.3%)
Past-6-month opioid/stimulant	391 (28.4%)	152 (45.9%)	255 (43.3%)	187 (58.8%)	985 (37.7%)
Age—mean [SD]	36.6 [11.5]	37.7 [10.9]	36.7 [11.5]	35.7 [10.7]	36.7 [11.3]
Sex/gender—*n* (%) female	744 (54.0%)	214 (64.7%)	388 (65.9%)	217 (68.2%)	1563 (59.8%)
Race/ethnicity					
White	1046 (79.6%)	245 (78.3%)	433 (77.6%)	230 (76.9%)	1954 (78.7%)
Asian American/Pacific Islander	120 (9.1%)	18 (5.8%)	32 (5.7%)	23 (7.7%)	193 (7.8%)
Black	73 (5.6%)	28 (8.9%)	44 (7.9%)	25 (8.4%)	170 (6.8%)
Hispanic	53 (4.0%)	16 (5.1%)	27 (4.8%)	18 (6.0%)	114 (4.6%)
Other	22 (1.7%)	6 (1.9%)	22 (3.9%)	3 (1.0%)	53 (2.1%)
Education attainment					
< Highschool/GED	8 (0.6%)	6 (1.8%)	11 (1.9%)	8 (2.5%)	33 (1.3%)
Highschool/GED	325 (23.6%)	134 (40.5%)	211 (35.8%)	162 (50.9%)	832 (31.8%)
2- or 4-year degree	759 (55.1%)	156 (47.1%)	279 (47.4%)	120 (37.7%)	1314 (50.2%)
Advanced degree	285 (20.7%)	35 (10.6%)	88 (14.9%)	28 (8.8%)	436 (16.7%)
Employment (past-year)					
Full time/student	954 (69.3%)	200 (60.4%)	341 (57.9%)	171 (53.8%)	1666 (63.7%)
Part time	185 (13.4%)	64 (19.3%)	92 (15.6%)	47 (14.8%)	388 (14.8%)
Unemployed; looking for work	100 (7.3%)	22 (6.6%)	73 (12.4%)	43 (13.5%)	238 (9.1%)
Unemployed; not looking	125 (9.1%)	35 (10.6%)	64 (10.9%)	39 (12.3%)	263 (10.1%)
Disabled	13 (0.9%)	10 (3.0%)	19 (3.2%)	18 (5.7%)	60 (2.3%)
Below poverty line income	230 (16.7%)	63 (19.0%)	150 (25.5%)	98 (30.8%)	541 (20.7%)
FDA rural/urban continuum (2013)					
Large metro	786 (57.1%)	164 (49.5%)	296 (50.3%)	152 (47.8%)	1398 (53.5%)
Non-metro	200 (14.5%)	63 (19.0%)	100 (17.0%)	57 (17.9%)	420 (16.1%)
Small metro	391 (28.4%)	104 (31.4%)	193 (32.8%)	109 (34.3%)	797 (30.5%)

### Family History and Justice Involvement

We asked participants whether they were adopted as a minor, whether they were raised by their biological mother and/or father, and whether they had ever been incarcerated. Compared to the low-MI-SUD class, the low-divorce class (OR = 4.07), moderate class (OR = 2.51), and high class (OR = 7.12) were significantly more likely to endorse being raised without their biological father. The moderate class and high class were more likely than the low-MI-SUD class to endorse being adopted (moderate OR = 3.65, high OR = 4.91) and being raised without their biological mother (moderate OR = 2.16, high OR = 5.28). The low-divorce (OR = 3.25), moderate (OR = 2.61), and high (OR = 6.21) classes were more likely than the low-MI-SUD class to have ever been incarcerated.

### Past-Year Substance Use

**Fig. 2:**
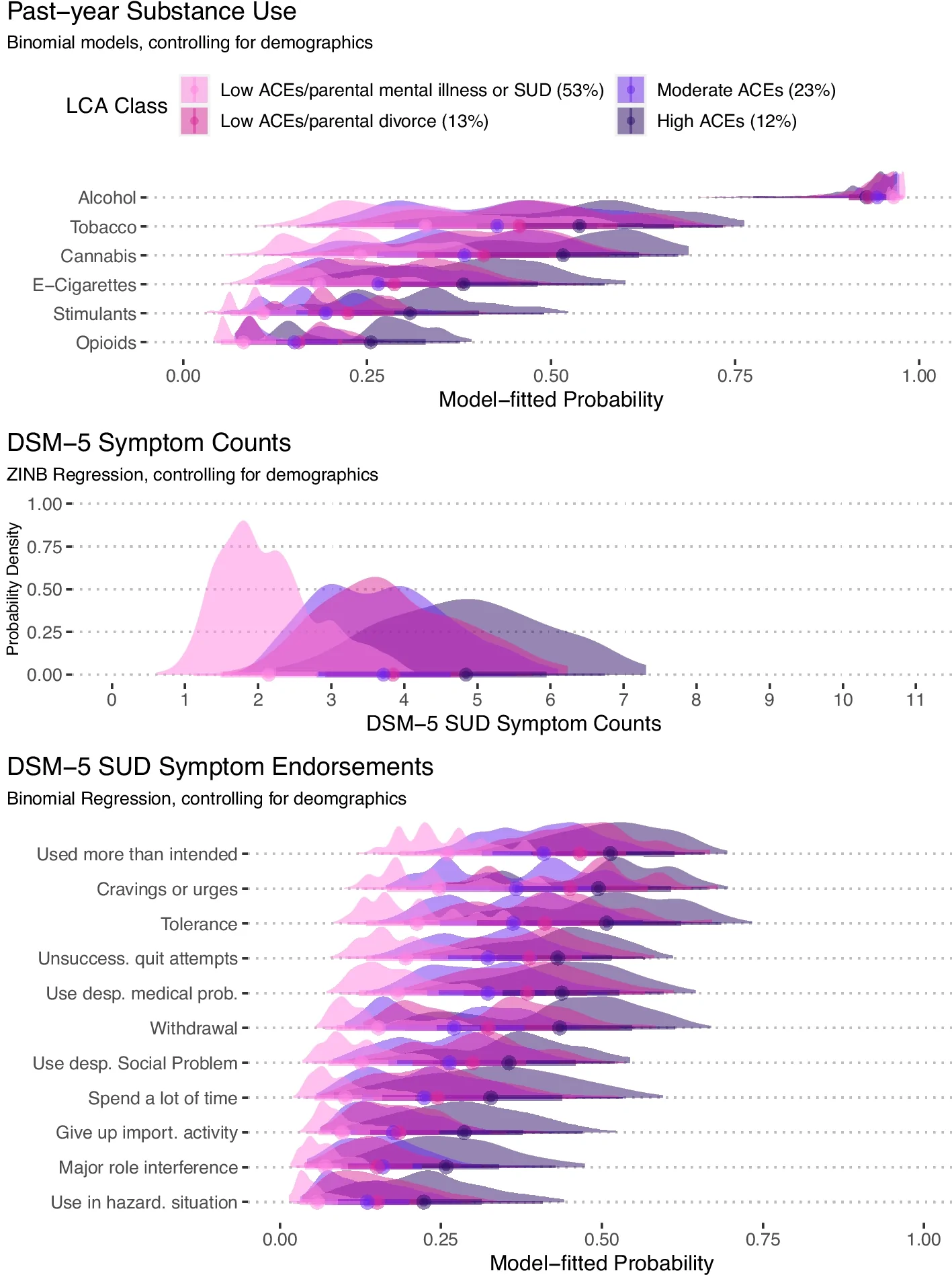
Outcomes for substance use. Top panel: LCA class probabilities of specific past-year substance use, estimated from binomial regression models. Middle panel: DSM-5 symptom counts, estimated from zero-inflated negative-binomial regression. Bottom panel: LCA class probabilities of endorsing individual DSM-5 SUD checklist items, estimated from binomial regression models. All models controlled for demographics and perceived childhood socioeconomic status

All classes displayed > 90% rates of past-year alcohol use (Fig. 2, top panel; Table 2). Compared to the low-MI-SUD class, the other three classes were all significantly more likely to report the use of cannabis (OR = 1.85–2.96), tobacco (OR = 1.46–2.12), e-cigarettes (OR = 1.49–2.37), opioids (nonmedically) (OR = 1.68–2.87), and stimulants (nonmedically) (OR = 1.77–3.03). The high ACEs class displayed the greatest likelihood of all examined substance classes (Fig. 2, top panel).

**Table 2:** Between-class differences in past-12-month substance use, estimated using binomial regression, controlling for demographic factors and perceived childhood socioeconomic status

Substance response variable	Between class contrasts			Ps-R2
	Low-MI-SUD—> low-divorce	Low-MI-SUD—> moderate	Low-MI-SUD—> high	
Past 6 months Opioid/stimulant Group^†^	2.01 *** [1.56, 2.60]	1.88 *** [1.52, 2.32]	3.38 *** [2.57, 4.46]	0.12
Alcohol	0.58 * [0.34, 0.98]	0.71 [0.44, 1.15]	0.66 [0.37, 1.16]	0.05
Cannabis	1.98 *** [1.52, 2.59]	1.85 *** [1.48, 2.30]	2.96 *** [2.24, 3.91]	0.12
Tobacco	1.57 *** [1.21, 2.03]	1.46 *** [1.18, 1.81]	2.12 *** [1.61, 2.80]	0.11
E-cig	1.61 ** [1.21, 2.14]	1.49 ** [1.17, 1.90]	2.37 *** [1.78, 3.17]	0.09
Nonmedical opioids	1.70 ** [1.17, 2.46]	1.68 *** [1.24, 2.29]	2.87 *** [2.03, 4.05]	0.09
Nonmedical Stimulants	2.15 *** [1.55, 2.97]	1.77 *** [1.34, 2.33]	3.03 *** [2.20, 4.17]	0.1

Standard errors are heteroskedasticity robust. *** *p* < 0.001; ** *p* < 0.01; * *p* < 0.05

^†^A study-design-specific classification, included in the model only as a covariate, not of direct interest for the present analyses

### Substance Use Disorder (SUD)

We examined between-class differences in perceived substance-related problems in the past year (yes/no), history of treatment for substance-use problems, past-year SUD symptom counts, and specific SUD symptoms. Compared to the low-MI-SUD class, the low-divorce (OR = 2.29), moderate (OR = 1.90), and high (OR = 2.67) classes were all more likely to endorse having a problem with one or more substances in the past year. Similarly, low-divorce (OR = 2.40), moderate (OR = 2.57), and high (OR = 3.24) classes were all more likely than low-MI-SUD to meet criteria for a past-year SUD. Compared to the low-MI-SUD class, the low-divorce (OR = 2.90), moderate (OR = 2.15), and high (OR = 5.67) classes were all more likely to report having received treatment for an SUD in their lifetime.

We modeled SUD symptom counts using zero-inflated negative-binomial regression, for which fitted values are displayed in the middle panel of Fig. 2. Low-divorce (OR = 0.49), moderate (OR = 0.39), and high (OR = 0.34) classes were all less likely to be classified as inflated zeros (i.e., displaying no SUD symptoms) than low-MI-SUD, and for those not classified as inflated zeros, low-divorce (IRR = 1.35), moderate (OR = 1.27), and high (OR = 1.55) classes all displayed higher SUD counts than low-MI-SUD.

Estimated probabilities of individual SUD symptoms are in the bottom panel of Fig. 2. The same pattern of results was observed for all individual symptoms as for past-year criteria and symptom counts, with low-divorce, moderate, and high all displaying greater endorsement odds than low-MI-SUD.

### Psychosocial Measures

These results are shown in Fig. S1. Controlling for demographic factors, we found that, compared to the low-MI-SUD class, the low-divorce (*b* = 1.29, partial-*R*^2^ = 0.01), moderate (*b* = 3.46, partial-*R*^2^ = 0.07), and high (*b* = 5.06, partial-*R*^2^ = 0.10) classes all scored higher in depressive symptom (CESD-10-R). Relative to the low-MI-SUD class, the low-divorce (*b* = 1.03, partial-*R*^2^ = 0.01), moderate (*b* = 2.51, partial-*R*^2^ = 0.05), and high (*b* = 3.92, partial-*R*^2^ = 0.08) classes all scored higher in anxiety symptom (GAD-7). Compared to the low-MI-SUD class, the low-divorce (*b* = 2.42, partial-*R*^2^ = 0.01), moderate (*b* = 6.62, partial-*R*^2^ = 0.06), and high (*b* = 8.02, partial-*R*^2^ = 0.06) classes all scores higher on the UCLA loneliness measure. Finally, relative to the low-MI-SUD class, the low-divorce (*b* = 1.33, partial-*R*^2^ = 0.00), moderate (*b* = 3.51, partial-*R*^2^ = 0.04), and high (*b* = 5.17, partial-*R*^2^ = 0.05) classes all scored higher on current perceived stress (PSS).

We examined these psychosocial measures as possible moderators of the association between ACE class and past-year SUD. There were differences in the time frame of assessment (past-year SUD versus past-month or past-2-week psychological problems), so these analyses do not permit conclusions about temporal order; we intended them only as a check for associations among measures of current adulthood functioning. After adjustment for familywide error protection, we found no associations between ACE class and SUD that were conditional on between-class differences in depressive symptoms (CES-D-R-10 scores), anxiety symptoms (GAD-7 scores), loneliness (UCLA Loneliness Scale scores), or general psychological distress (PSS scores).

## Discussion

### Latent Classes of ACEs

In a nationwide online sample of US adults who use alcohol or other substances, with reported use patterns ranging from nonproblematic to SUD, we identified four latent classes of ACE history: 1) low except parental divorce (low-divorce), 2) low except exposure to mental illness or SUD (low-MI-SUD), 3) moderate, and 4) high. The use of LCA in ACE analyses allows for a holistic assessment of patterns of co-occurrence that may otherwise be hidden (Brown et al., 2019; Lanier et al., 2018). Latent classes are created from patterns of shared ACEs in the sample, irrespective of a priori divisions of ACEs into conceptual domains (physical abuse, emotional abuse, and so on). We could not know in advance whether the classes would reflect the a priori domains, or alternatively, whether they would merely reflect differences in overall amount of event exposure. We found that they mostly did reflect differences in amount of exposure, but event-specific differences became salient in the two classes with lower overall exposure. The a priori domains did not distinguish the classes. Thus, the four classes represent intradomain co-occurrence of ACEs, consistent with findings from other LCA studies of ACEs (Brown et al., 2019; Scott et al., 2013).

### Group-Level Relationships Between ACE Exposure and Adult Outcomes: Reliable, but not Straightforward

We looked for associations between class membership and adult psychosocial measures (anxiety, depression, stress, and loneliness) and patterns of substance use. Membership within the high ACE class was associated with higher likelihoods of past-year substance use, past-year problematic use, and SUD symptomatology. Membership within the high class was also related to increased likelihood of psychosocial problems. All psychosocial measures along with past-year problematic use and SUD symptomatology exhibited a dose–response relationships with ACEs exposure, but the substance-use measures did not: the moderate class was less likely to use than the low-divorce class across all substances, suggesting that substance-related outcomes cannot be assumed to be a straightforward reflection of amount of ACE exposure.

Substance-related outcomes also showed an unexpected relationship with specific patterns of ACE exposure: membership in our low-MI-SUD class was not associated with greater likelihood of problematic substance use, lifetime SUD treatment, SUD symptoms, or past-year SUD diagnosis. This may appear to be a departure from theories of transgenerational addiction, which posit genetics, epigenetic inheritance, and environmental exposure as mechanisms of concentration of addiction within families (Merikangas & McClair, 2012). For some people, witnessing problematic substance use in parents is a protective factor against problematic substance use because they purposefully avoid or limit their own substance use, a phenomenon called aversive transmission (Georgeson et al., 2024). Aversive transmission may help explain this unexpected aspect of our findings, though we cannot be sure because we did not specifically assess it. An opportunity for future research would be to do so, such that firmer conclusions can be drawn from unexpected findings.

Trajectories after exposure to ACEs may be considerably worse in places where children have undergone massive displacements (Düken & Kaplan, 2024), especially where there are fewer systems to prevent them from having to grow up on the street (Kaplan & Çuhadar, 2020) or where there are significant war-related events and socio-political instabilities (Naal et al., 2018). ACE/outcome studies are fewer in developing nations, but research is in progress in several such nations, with results suggesting both cross-cultural commonalities and culture-specific differences. The findings in our sample will need to be tested for replicability in other contexts, which may be done using a version of the ACEs (*Adverse Childhood Experiences International Questionnaire (ACE-IQ*, n.d.) that was developed by the World Health Organization for global use, incorporating assessment of large-scale social disruptions as well as familial disruptions.

### Individual-Level Relationships Between ACE Exposure and Adult Outcomes: Not Reliable

At least as striking as our group-level findings was our finding of heterogeneity within each ACE class, reflected in modest *R*^2^ values, and best seen in the spread of individual data points (Fig. S1 bottom panel for psychosocial measures; Fig. S2 for substance use and SUDs). This raises an issue that is not specific to ACEs: group-level associations described with inferential statistics can help support mechanistic explanations of behavior, but are rarely suitable for predicting individual instances of behavior; the latter endeavor can require different statistical approaches and different ways of expressing accuracy, emphasizing rates of false positives and negatives (Yarkoni & Westfall, 2017). People in clinical settings are sometimes screened for ACEs in an effort to target interventions toward them, but these screens are not sufficiently accurate for that purpose (Baldwin & Danese, 2021). Identifying children as having experienced multiple adversities may label them as “high-risk” for downstream psychological and physical health problems. This can create an “expectancy effect,” where parents, clinicians, and teachers notice abnormal behavior in a way that reinforces their predictions of unfavorable outcomes for these children (Campbell, 2020). It is advisable, then, to exercise caution in making case-level clinical decisions from extrapolation of research findings on ACE/outcome associations. In their scoping review of ACE studies using LCA, Wang et al. (2024) emphasize inconsistencies that accompany differences across studies with regard to sample sizes, ACE questionnaires, and the numbers and types of ACE indicators used for LCA analysis. Our findings show that even within one study, using one sample, heterogeneity is to be expected, and prediction is perilous.

It is especially striking that the degree of case-by-case variability in our sample was high because we assessed both ACEs and adult outcomes retrospectively and cross-sectionally, an approach that actually maximizes the observed association between the two (Baldwin et al., 2024; Danese & Widom, 2020; Widom et al., 1999). We found group-level ORs across all psychosocial measures (1.315 to 8.743) that were comparable to those seen in other retrospective studies of ACEs and adult outcomes, but the group-level ORs do not speak to the accuracy of person-level classification or prediction.

A relevant cautionary tale is the use of one type of ACE for prediction of the likelihood of opioid misuse or opioid use disorder (OUD) in people being evaluated for opioid prescriptions for pain. In the original version of a questionnaire called the Opioid Risk Tool (ORT), risk of opioid misuse or OUD was scored higher for women who reported any history of preadolescent sexual abuse; the scoring rubric was based on group-level associations, but was used to deny prescriptions to individual women (Webster & Webster, 2005). This use of the ORT was criticized by patient and provider communities and by one of the authors of the ORT (Anson & Webster, 2019; Schechtman & Szalavitz, 2019). A 2019 study found that the sexual-abuse item was not reliably predictive even at the group level, and the item was removed from a revised (Cheatle et al., 2019)—yet the original version of the ORT continued to be administered in some clinics and may still be used to deny opioid pain medication to women with histories of sexual abuse (Anson & Webster, 2019; Schechtman & Szalavitz, 2019). Our findings add to the body of literature that militates against the misreading of group-level findings (whether or not they subsequently replicate) as algorithms for individual case management.

There may still be a place for clinical assessment of ACEs. As noted above, social disruptions and their pernicious effects may occur at large scales as well as intrafamilial scales. Clinicians could assess ACEs in their patients and generate regularly updated summary reports at the level of whole practices, to be compiled and monitored by health and social-welfare agencies at the level of a whole community or region. Changes in aggregate exposure to ACEs could then be used to scale up the availability (and publicizing) of resources for children needing housing or care and for anyone needing support for ongoing effects of prior exposures. Such a system would avoid the pitfalls of mislabeling any one patient, while shoring up protections for anyone in the community who experiences problems from ACEs.

### Limitations

One limitation of our study is that we assessed ACEs through retrospective self-report only, which may result in potential inaccuracies through recall bias. As noted, however, this makes the heterogeneity in our results all the more striking. Second, as with any use of LCA, correct class assignment for every participant is not guaranteed. Third, we used an online sample from a specific platform, not a probability sample, and some groups were underrepresented, most notably Black women. For this reason, and as noted in the introduction by the inclusion of studies from different sociocultural contexts, our US-based sample has limited generalizability.

To date, there are no additional open-source software updates that can improve upon the statistical modeling conducted in our analyses. Advances in closed-source LCA software provide additional modeling capabilities applicable to covariate model construction and analyses of longitudinal datasets, but are outside of the scope of this study (Depaoli et al., 2025; Vermunt & Magidson, 2021).

## Conclusions

Using LCA, we showed that specific patterns of ACE exposure (such as exposure to parental SUDs) were differentially informative about adult outcomes. This finding contrasts with a prior finding that ACE exposure could be adequately described by simply cumulating exposure types in a score, an approach that would obviate LCA (Merians et al., 2019). The difference in findings may reflect differences across samples or across outcome assessments, but it underscores the importance of checking whether LCA adds information about ACE effects, as it did here.

At the same time, our findings underscore the need for caution in discussing the predictive power of ACEs for poorer health outcomes, most specifically depression, anxiety, loneliness, stress, and substance use patterns and disorders. Even though we assessed ACEs through retrospective self-report (the approach that typically produces the strongest associations with current problems), we found substantial heterogeneity in all associations we tested, indicating that ACEs were highly fallible predictors of well-being, substance use, and substance-use problems at the level of individual respondents. Future work could explore resilience, although resilience itself is a fraught concept, sometimes displacing responsibility from social systems to affected individuals (Suslovic & Lett, 2024). Resilience may also be short lived: a longitudinal study of the Great Smoky Mountains cohort found that youth exposed to multiple adversities, who displayed no psychiatric problems in childhood, later experienced significantly higher rates of anxiety, depressive disorders, poorer physical health, and educational/financial functioning in adulthood than children exposed to fewer ACEs. These findings underscore the importance of preventing childhood adversity rather than relying on resilience alone (Copeland et al., 2023).

## Supplementary Information

Below is the link to the electronic supplementary material.

ESM 1 (PDF 3.06 MB)

ESM 2 (PDF 3.06 MB)
