# Supplementary material for: Latent Classes of Adverse Childhood Experiences (ACEs) and Adult Substance-use Problems and Psychosocial Outcomes: Complex and Heterogeneous Associations
Source: Int J Ment Health Addict. 2025 Aug 6;24(4):2866–85. doi: 10.1007/s11469-025-01527-w (PMC13499878; doi:10.1007/s11469-025-01527-w)
Supplement: Supplementary file 1 [file 11469_2025_1527_MOESM1_ESM.pdf]

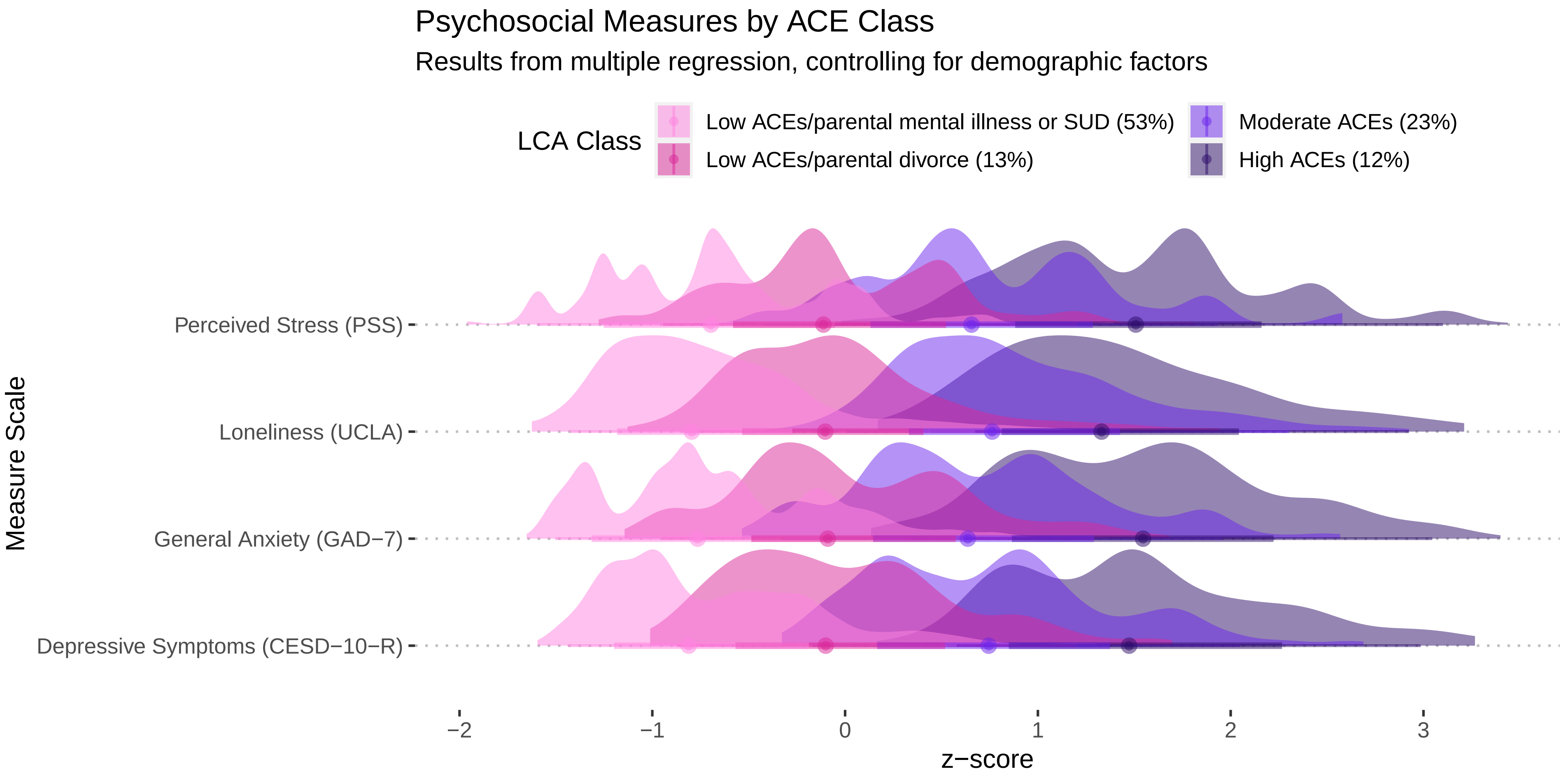
Psychosocial Measures by ACE Class
Results from multiple regression, controlling for demographic factors
LCA Class
Low ACEs/parental mental illness or SUD (53%)
Low ACEs/parental divorce (13%)
Moderate ACEs (23%)
High ACEs (12%)
Measure Scale
Perceived Stress (PSS)
Loneliness (UCLA)
General Anxiety (GAD−7)
Depressive Symptoms (CESD−10−R)
−2
−1
0
1
2
3
z−score

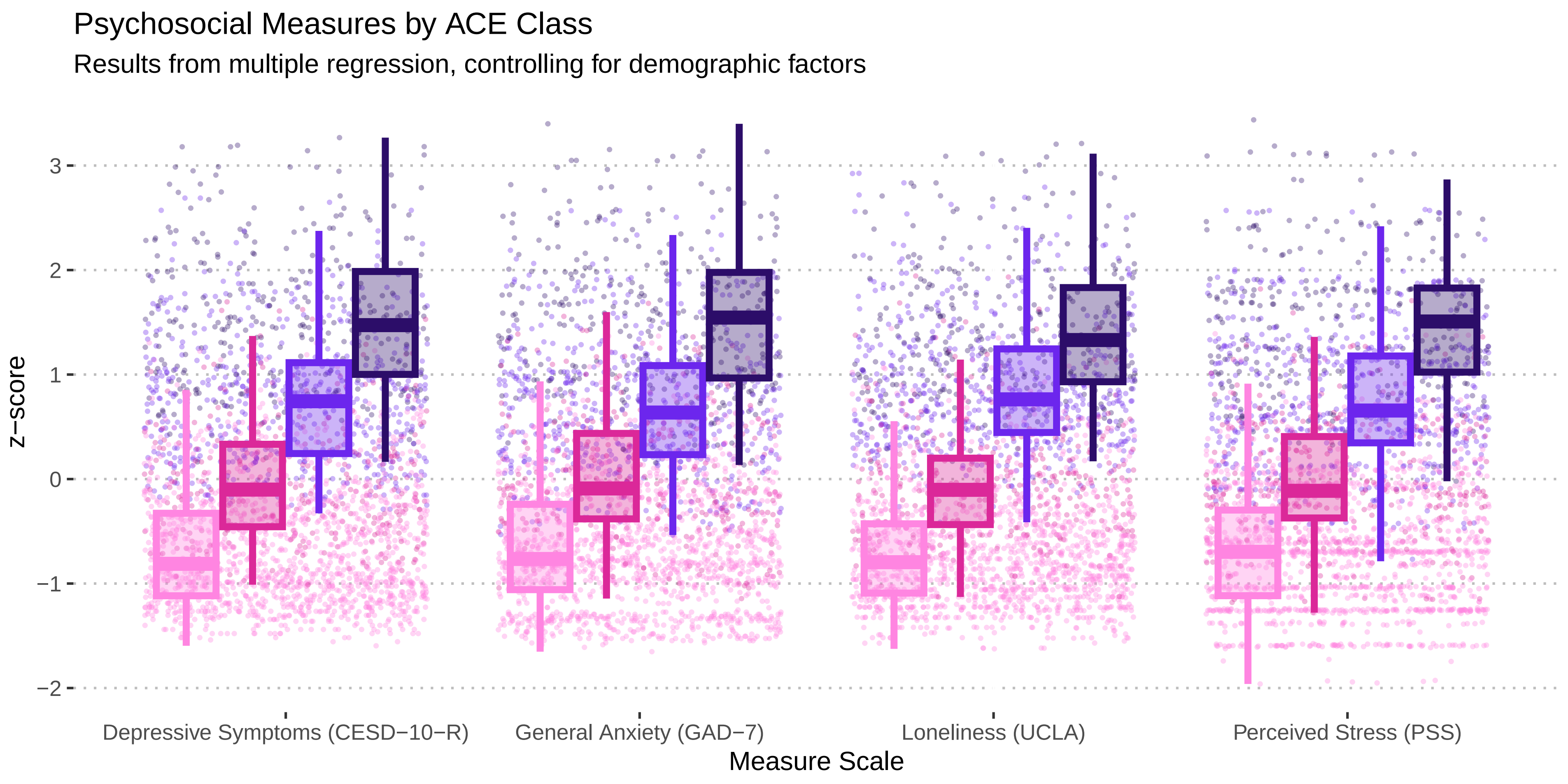
Psychosocial Measures by ACE Class
Results from multiple regression, controlling for demographic factors
z−score
3
2
1
0
−1
−2
Depressive Symptoms (CESD−10−R)
General Anxiety (GAD−7)
Loneliness (UCLA)
Perceived Stress (PSS)
Measure Scale
