# Supplementary material for: Latent Classes of Adverse Childhood Experiences (ACEs) and Adult Substance-use Problems and Psychosocial Outcomes: Complex and Heterogeneous Associations
Source: Int J Ment Health Addict. 2025 Aug 6;24(4):2866–85. doi: 10.1007/s11469-025-01527-w (PMC13499878; doi:10.1007/s11469-025-01527-w)
Supplement: Supplementary file 2 [file 11469_2025_1527_MOESM2_ESM.pdf]

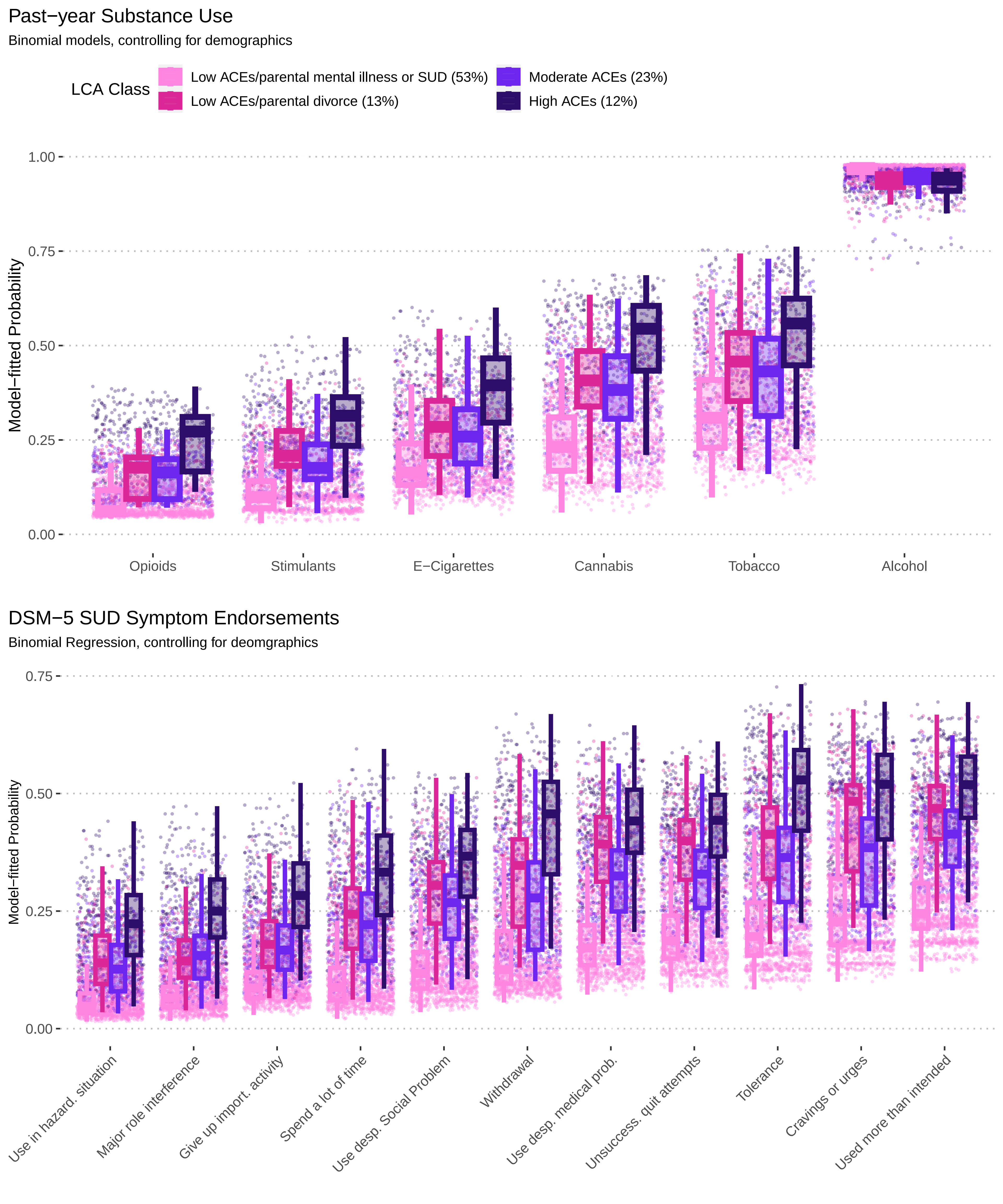
Past−year Substance Use
Binomial models, controlling for demographics
LCA Class
Low ACEs/parental mental illness or SUD (53%)
Low ACEs/parental divorce (13%)
Moderate ACEs (23%)
High ACEs (12%)
Model−fitted Probability
1.00
0.75
0.50
0.25
0.00
Opioids
Stimulants
E−Cigarettes
Cannabis
Tobacco
Alcohol
DSM−5 SUD Symptom Endorsements
Binomial Regression, controlling for deomgraphics
Model−fitted Probability
0.75
0.50
0.25
0.00
Use in hazard. situation
Major role interference
Give up import. activity
Spend a lot of time
Use desp. Social Problem
Withdrawal
Use desp. medical prob.
Unsuccess. quit attempts
Tolerance
Cravings or urges
Used more than intended
